# Responding to GPs' information resource needs: implementation and evaluation of a complementary medicines information resource in Queensland general practice

**DOI:** 10.1186/1472-6882-11-77

**Published:** 2011-09-20

**Authors:** Tina Janamian, Stephen P Myers, Peter O'Rourke, Heather Eastwood

**Affiliations:** 1 Discipline of General Practice, University of Queensland, Brisbane, Australia; 2 NatMed-Research Unit, Southern Cross Plant Science, Southern Cross University, Lismore, Australia; 3 Queensland Institute of Medical Research, Brisbane, Australia; 4 Medical Education Services Australia (MESA), Notting Hill, Australia

## Abstract

**Background:**

Australian General Practitioners (GPs) are in the forefront of primary health care and in an excellent position to communicate with their patients and educate them about Complementary Medicines (CMs) use. However previous studies have demonstrated that GPs lack the knowledge required about CMs to effectively communicate with patients about their CMs use and they perceive a need for information resources on CMs to use in their clinical practice. This study aimed to develop, implement, and evaluate a CMs information resource in Queensland (Qld) general practice.

**Methods:**

The results of the needs assessment survey of Qld general practitioners (GPs) informed the development of a CMs information resource which was then put through an implementation and evaluation cycle in Qld general practice. The CMs information resource was a set of evidence-based herbal medicine fact sheets. This resource was utilised by 100 Qld GPs in their clinical practice for four weeks and was then evaluated. The evaluation assessed GPs' (1) utilisation of the resource (2) perceived quality, usefulness and satisfaction with the resource and (3) perceived impact of the resource on their knowledge, attitudes, and practice of CMs.

**Results:**

Ninety two out of the 100 GPs completed the four week evaluation of the fact sheets and returned the post-intervention survey. The herbal medicine fact sheets produced by this study were well accepted and utilised by Qld GPs. The majority of GPs perceived that the fact sheets were a useful resource for their clinical practice. The fact sheets improved GPs' attitudes towards CMs, increased their knowledge of those herbal medicines and improved their communication with their patients about those specific herbs. Eighty-six percent of GPs agreed that if they had adequate resources on CMs, like the herbal medicine fact sheets, then they would communicate more to their patients about their use of CMs.

**Conclusion:**

Further educational interventions on CMs need to be provided to GPs to increase their knowledge of CMs and to improve their communication with patients about their CMs use.

## Background

The Australian community are substantial users of complementary medicines (CMs) [1,2] with more than half of the population using them in some form, mainly self-initiated herbal medicines and vitamins and minerals. Given that the therapeutic efficacy of some of these preparations has first class evidence of efficacy [3-5], some Australian GPs have included their use in clinical practice [6]. We have recently demonstrated however, that majority of GPs lack knowledge and require information on CMs [7]. Even those GPs that are sceptical of CMs need to be aware of the potentially harmful effects of certain CMs approaches [8], in particular herbal medicines, as well as the potentially harmful effects of concurrent use of specific pharmaceutical CMs combinations [9]. It is important that the Australian community, including health professionals, are educated about these modalities. In particular, recent studies [7,10] have highlighted concerns over the lack of communication between primary health care physicians and their patients regarding CMs.

As a health professional group, Australian GPs are in the forefront of primary health care and public health and are in an excellent position to communicate with their patients and educate them about CMs [11] provided that they have appropriate scientifically validated up to date information to base these communications. To be able to provide information on CMs to patients, GPs will need a working knowledge of the subject and become informed about different CMs modalities [11-14]. Physicians cannot become knowledgeable about all CMs practices, but they can apply the principles of evidence-based medicine, as in any area of health care [14]. At the very least, GPs will need understanding of CMs benefits, side effects, interactions, issues of contamination, and recommended dosages [12,15]. Undergraduate, graduate clinical, and continuing education could play an important role in informing GPs with up-to-date and relevant information. GPs also need to be provided with appropriate evidence-based information resources to use in their clinical practice [7,16].

We have demonstrated that GPs lack the knowledge required about CMs and this study set out to develop a CMs information resource based on the findings of our needs assessment survey [7], and to implement and evaluate the CMs information resource in Qld general practice. It was hypothesised that the provision of an appropriate CMs information resources would be well received by GPs, and was likely to positively impact GPs' attitude, knowledge and practice of CMs. It was also hypothesised that having appropriate CMs information resources would help improve GPs knowledge of CMs, give them more confidence and improve their communication with their patients about CMs.

## Methods

Ethics approval for this study was obtained through the University of Queensland's Medical Research Ethics Committee (Approval Number 2002000194).

### Resource development

The findings of a needs assessment survey of Queensland (Qld) general practitioners were used to inform development of a CMs information resource for GPs to use in their clinical practice [7]. The needs assessment survey indicated that fact sheets were GPs' most preferred type of information resource for receiving CMs information to use in clinical practice. GPs equally perceived most need for an information resource on herbal medicines or vitamins, minerals, and trace elements. It was decided to focus the information resource on herbal medicines. The majority of GPs wanted the information resource to contain evidence-based literature (87%), pharmacological (80%) and toxicological information (66%). Based on this information it was decided that a set of herbal medicine fact sheets containing evidence-based literature, pharmacological, and toxicological information would be developed.

The time frame of the study permitted the development and production of 4 herbal medicine fact sheets. The 4 herbal medicines selected for the fact sheets were St John's wort, ginkgo biloba, saw palmetto and valerian. These were selected based on a review of literature of commonly used herbal medicines in Australia [17-21], review of the scientific evidence available on herbal medicines, and consultation with the study expert advisory group (a panel of nine, including local GPs, GPs with CMs qualifications or experience, a sociologist with expertise in the field of CMs and a lecturer in needs assessment methodology). Each fact sheet is a double-sided A4 sheet, with the front side containing information on the herb including clinical uses, major chemical components, pharmacological actions, dosage and administration, adverse effects, most common adverse effects, rare adverse effects, contraindications and drug interactions. The reverse side includes evidence-based literature and results of the latest clinical trials on the specific herb, with a short summary of the evidence and references for the clinical trials. The content, layout and format of the fact sheets was informed by existing resources on CMs for GPs and health professionals including reputable databases (Table 1). The 4 fact sheets used are given in an additional file (Additional file 1).

**Table 1:** Sources used for informing herbal medicines information fact sheets.

Source	Name of Source
**Databases Used**	• The Natural Standard Professional Database is an international research collaboration that aggregates and synthesizes data on complementary and alternative therapies, http://www.naturalstandard.com
	• Integrative Medicine Gateway - Australian based web site that provides access to scientific information, education and communication about Integrative Medicine http://www.imgateway.net/page.jsp?p_name=Home
	• Natural Medicines Comprehensive Database - Scientific Gold standard evidence-based reviews of more than 1000 herbal medicines. http://www.naturaldatabase.com
	• National Centre for Complementary and Alternative Medicine - US government site both for consumers and healthcare professionals http://nccam.nih.gov/
	• Alternative Medicine Foundation (HerbMed) - an interactive, electronic herbal database http://www.herbmed.org
	• Bandolier Complementary and Alternative Therapies - contains systematic reviews and meta-analyses on effectiveness of complementary medicines http://www.medicine.ox.ac.uk/bandolier/
	• WHO monographs - contains monographs of herbal medicines http://www.who.int/en/
	• Cochrane Collaboration Database of Systematic Reviews - contains reports of randomised controlled trials and systematic reviews on complementary medicine.
	• Altmedex - contains over 140 monographs on herbal medicines and is updated regularly.
	• Medline - Abstracts from numerous medical journals including CM speciality journals
	• PubMed - The National Centre for Complementary and Alternative Medicine and The National Library of Medicine have partnered to create CAM on PubMed, http://nccam.nih.gov/research/camonpubmed/
	• Allied and Complementary Medicine Database (AMED) - database produced by the British Library's Medical information centre in the UK.

**Books Used**	• Ernst E. *The desk-top guide to complementary and alternative medicine - an evidence-based approach*. St Louis: Mosby, 2001.
	• Fugh-Berman A. *The 5-minute herb and dietary supplement consult*. Philadelphia: Lippincott Williams and Wilkins, 2003.
	• Blumenthal M. *The ABC clinical guide to herbs*. Austin, US: American Botanical Council, 2003.
	• Fetrow C, Avila J. *Professional's handbook of complementary and alternative medicines*. Second edition. Springhouse: Springhouse Corp, 2001.
	• Bascom A. *Incorporating herbal medicines into clinical practice*. Philadelphia:F.A. Davis Company, 2002.
	• Barnes J, Anderson L, Phillipson J. *Herbal medicines - a guide for healthcare professionals*. Second edition. London: Pharmaceutical Press, 2002.
	• Fleming T. *PDR for herbal medicines*. Second edition. Montvale, US: Medical Economics Company, 1998.

### Implementation and evaluation

The facts sheets were mailed to self-identified GPs in 100 Qld general practices to trial for 4 weeks. These GPs had indicated in a previous needs assessment survey that they would trial the facts sheets in their practice. Verbal consent was obtained before the fact sheets were mailed out to GPs. The cover letter that accompanied the fact sheets informed GPs that they could withdraw from the study at any time. GPs were asked to use the fact sheets as many times as possible and whenever they felt appropriate (with or without a patient present). Follow-up phone calls were made to each practice during the trial period to confirm receipt of fact sheets, to monitor progress, and provide any support. A post-intervention evaluation questionnaire was mailed to participating GPs at the end of the 4 week trial period to determine GPs' acceptance and utilisation of the fact sheets, their satisfaction with the overall quality, content, presentation, and practicality of the resource, the impact of the resource on GPs' perceived attitudes towards CMs, their knowledge of CMs, and their practice of CMs, in particular the herbal medicines covered in the fact sheets. Four follow up reminders via phone and fax were conducted to achieve a high response rate. The questionnaire data was analysed using Statistical Package for the Social Sciences (SPSS) version 10.0.

## Results

### Response rate

The overall response rate for returned post-intervention surveys by GPs was 92 (92%). Reasons for not responding included illness, on long term leave or on holidays, changed practice or too busy.

The representative nature of the respondents was compared to the GP population in Australia [22] and the GP population in Qld using the Bettering the Evaluation And Care of Health (BEACH) survey of general practice activity 1998-2003 [23] (Table 2). The respondents were over-representative of female and younger GPs compared to the GP population in Australia and Qld. The respondents were under-representative of the rural/remote GPs.

**Table 2:** Characteristics of respondents to the evaluation with GPs in Australia and Qld (%).

GP demographics	**BEACH Study 1998-2003 Australian sample **[23] (n = 5,021)	**BEACH study 1998-2003 Qld sample **[23] (n = 933)	**GP population Australian sample **[22] (n = 18,787)	Respondents (n = 92)
**Gender**				
Male	67.4	64.6	66	58.1
Female	32.6	35.4	34	41.9
**Age distribution**				
<34	7.2	7.1	12	14.1
35-44	30.1	33.9	32	48.8
45-44	34.2	33.2	30	26.7
65+	28.4	25.8	26	10.5
**Practice location**				
Capital city	67.1	50.6	68	47.7
Other metropolitan	7.7	14.1	7	37.2
Rural/remote	25.3	35.4	25	15.2

### Post-intervention survey responses

#### Frequency of use

Six GPs indicated that they "never" used the herbal medicine fact sheets and these 6 were excluded from the remaining analysis (i.e. all percentages are given as the proportion of the 86 respondents who did use the fact sheets). A third of the GPs had used the herbal medicine fact sheets at least once, a third between one and four times and the remaining third (32%) had used them 4 or more times.

When GPs were asked "did you use the herbal medicine fact sheets as many times as you could have", 45% of respondents answered yes. The most common reasons for not doing so were that they forgot (33%), not enough time (19%), and being too busy (15%). The majority of GPs (79%) used the St Johns Wort fact sheet, 57% used the valerian fact sheet, 54% used the ginkgo fact sheet, and 45% used the saw palmetto fact sheet. About 20% of respondents had used all 4 fact sheets in the 4 week period. This was supported by the majority of GPs reporting that they frequently saw patients with depression (73%) and insomnia (63%), occasionally seeing patients with benign prostatic hypertrophy (40%), dementia or cerebral insufficiency (42%), and intermittent claudication (69%).

#### Reasons for using or not using fact sheets

The most common reason for using a specific fact sheet was that the GP saw more patients with the right condition (59%), while 23% perceived the herb to be effective, 15% believed that the evidence for this herb is strong, and 35% indicated other reasons for using the herbal medicine fact sheets. Frequently listed reasons in the other category included patients asking about them and reading the fact sheets to increase own knowledge. Of the 6 GPs who indicated not using the fact sheets, 4 forgot, 1 said that they do not believe the herb is effective, 1 GP said they prefer to use the most effective drug available.

#### How GPs used the fact sheets in their clinical practice

GPs mainly used the fact sheets to increase their own knowledge (80%), answer patients' questions (71%), and advise patients (66%). Some GPs used the fact sheets to recommend or prescribe the herbs to patients (37%), and 49% showed the fact sheets to patients and 29% even made a copy of the fact sheets for patients.

#### GPs' assessment of the herbal medicine fact sheets (quality, usefulness, satisfaction)

The majority of the GPs agreed with the statements on their perception of the quality, usefulness of the fact sheets, and their satisfaction with the fact sheets (Table 3). For instance, 98% of GPs agreed that overall, the herbal medicine fact sheets contain good quality information and that overall the fact sheets are well presented, 95% agreed that the fact sheets are useful in providing them with the information that they need about the herbal medicines, and 92% agreed that overall they were satisfied with the assistance the fact sheets provided them to communicate with their patients.

**Table 3:** GPs' assessment of the herbal medicine fact sheets.

Statement	Disagree or Strongly Disagree	Neither Agree Or Disagree	Agree-Strongly Agee
**Quality (n = 85)**			

Overall, the herbal medicine fact sheets contain good quality information	0	2.3	**97.4**

The herbal medicine fact sheets have appropriate content	0	3.5	**96.5**

The herbal medicine fact sheets have sufficient information	1.2	5.8	**93.0**

The content in the herbal medicine fact sheets is well presented	0	4.7	**95.4**

The content is easy to read and comprehend	0	5.8	**94.2**

The fact sheets are easy to use in clinical practice	2.4	5.8	**91.9**

Overall the fact sheets are well presented	0	2.3	**97.7**

**Usefulness (n = 86)**			

The fact sheets are useful in providing me with the information that I need about the herbal medicines	1.2	3.5	**95.3**

The herbal medicine fact sheets were useful in assisting me to communicate (e.g. discuss, advise, answer questions) information about herbal medicines with my patients	0	12.8	**87.2**

The herbal medicine fact sheets are a useful resource for my clinical practice	0	4.7	**95.3**

**Satisfaction (n = 86)**			

Overall I am satisfied with the assistance the fact sheets provided me to communicate with my patients	1.2	7.0	**91.9**

I am satisfied with the content of the information in the fact sheets	1.2	5.8	**93.1**

I am satisfied with the appearance of the fact sheets	0	2.3	**97.7**

I am satisfied with the practicality of the fact sheets to use in my clinical practice	4.7	9.3	**86.0**

I am satisfied with the confidence the fact sheets gave me to communicate with my patients about their use of herbal medicine	2.3	9.3	**88.4**

#### Perceived impact on knowledge of CMs, attitudes towards CMs, and clinical practice of CMs

Table 4 includes the results of the impact of the fact sheets on GPs' perceived knowledge of CMs, attitudes towards CMs, and clinical practice of CMs. Sixty six percent of GPs agreed that prior to receiving the fact sheets they felt they did not have adequate knowledge to discuss those herbal medicine options with their patients. Approximately 90% of GPs agreed that overall the fact sheets have increased their knowledge of herbal medicines, and provided them with adequate knowledge to be able to communicate with patients about those specific herbs and reassured them that the information they were conveying to patients was correct.

**Table 4:** GPs' perception on usage of the herbal medicine fact sheets and its impact on their knowledge, attitude and clinical practice (n = 86).

Statement	Disagree or Strongly Disagree	Neither Agree Or Disagree	Agree-Strongly Agee
**Impact on knowledge**			

Prior to receiving the herbal medicine fact sheets I felt that I did not have adequate knowledge to discuss those herbal medicine options with my patients	14.0	19.8	66.3

The herbal medicine fact sheets have provided me with adequate knowledge to be able to communicate with patients about specific herbs	0.0	10.5	89.5

Having the herbal medicine fact sheets reassured me that the information I was conveying to patients was correct	0.0	14.0	86.3

Overall the herbal medicine fact sheets have increased my knowledge of herbal medicines	1.2	7.0	91.8

**Impact on attitude**			

Since using the resource, I feel more positive discussing herbal medicines with my patients	2.3	24.4	73.2

Having the herbal medicine fact sheets makes me feel more confident discussing herbal medicines with my patients	2.3	12.8	84.9

Since using the herbal medicine fact sheets, I feel more positive in general about complementary medicine	14.0	40.7	45.3

**Impact on clinical practice behaviours**			

During the last four weeks, I questioned my patients about their herbal medicine use more often than I usually would have questioned them	29.9	36.0	33.7

During the last four weeks, I discussed herbal medicine options with my patients more often than I usually would have discussed them	29.1	34.9	36.1

During the last four weeks, I recommended one or more of the herbal medicines mentioned in the fact sheets to the patients with the right conditions more often than I usually would have recommended them	27.9	29.1	43.0

Since having the herbal medicine fact sheets I would now consider using other clinically proven complementary medicine options for my patients	12.8	27.9	59.4

If I had adequate resources on complementary medicine, like the herbal medicine fact sheets, then I would communicate (question/discuss/recommend) more to my patients about their use of complementary medicine	2.3	11.6	86.1

The majority of GPs (73%) agreed that since using the resource, they feel more positive discussing herbal medicines with their patients and 85% agreed that having the herbal medicine fact sheets makes them feel more confident discussing herbal medicine with my patients", and almost half of respondents agreed (45%) that since using the herbal medicine fact sheets, they feel more positive in general about complementary medicine.

About one third of responding GPs' agreed that they questioned their patients about their herbal medicine use and discussed herbal medicine options with their patients more often than they usually would have. Nearly half of the GPs agreed that they recommended one or more of the herbal medicines mentioned in the fact sheets to the patient with the right conditions more often than they usually would have recommended them. Interestingly nearly 60% of GPs agreed that since having the herbal medicine fact sheets, they would now consider using other clinically proven CMs options for their patients. Eighty six percent of GPs agreed that if they had adequate resources on CMs, like the herbal medicine fact sheets, then they would communicate more to their patients about CMs.

There were positive associations between GPs reasons for using fact sheet and how they used the fact sheet. GPs whose reason for using the fact sheets was that they believe the herb is effective were more likely (p < 0.001) to have used the fact sheets for recommending or prescribing the herb to patients (70.0%) compared to GPs who did not believe the herb is effective (27.3%). GPs who indicated reason for use as evidence is strong for this herb were also more likely (61.5%) to have used the fact sheets to recommend or prescribe to patients than GPs (32.9%) who did not believe the evidence is strong for this herb (p = 0.049). GPs who believe the herb is effective were also more likely (60.0%) to have given patients a copy of the fact sheets than GPs (19.7%) who did not believe the herb is effective (p < 0.001).

There was also some positive association between GPs perceived impact on knowledge of CMs and GPs' attitudes towards CMs and GPs' clinical practice of CMs. For instance, GPs who agreed to the knowledge statement "prior to receiving the herbal medicine fact sheets I felt that I did not have adequate knowledge to discuss those herbal medicine options with my patients" were more likely (p = 0.002) to agree to the statement "during the last four weeks, I questioned my patients about herbal medicine use more often than I usually would have questioned them". They were also more likely (p = 0.015) to agree to the statement "during the last four weeks, I recommended one or more of the herbal medicines mentioned in the fact sheets to the patients with the right conditions more often than I usually would have recommended them".

GPs who agreed to the attitude statement "since having the herbal medicine fact sheets, I feel more positive in general about complementary medicine" were more likely (p = 0.002) to agree to the statement "during the last four weeks, I questioned my patients about their herbal medicine use more often than I usually would have questioned them" than GPs who were equivocal or disagreed to the attitude statement. The other associations followed a similar trend.

#### Main strengths and shortcomings of the fact sheets and suggestion for improving fact sheets

GPs were asked to comment on the strengths of the fact sheets and more than half of responding GPs wrote a comment. The common themes mentioned were in regards to the appearance, content, and usefulness of the fact sheets. Some GPs also made general comments about the fact sheets (Table 5). Thirty five GPs made comments about the shortcomings of the fact sheets, most of these were related to having a computerised version of the fact sheet (Table 5). Twenty five GPs gave suggestions on ways to improve the fact sheets and these were related to the shortcomings, such as having a computerised version (n = 8), having more fact sheets on other commonly used herbs (n = 5), to have a folder or ring binder for all fact sheets (n = 5), have similar patient handouts (n = 3), and to laminate them (n = 2).

**Table 5:** GPs' qualitative comments regarding strength and shortcomings the herbal medicines fact sheets.

Strengths	Themes and comments
	Good size (n = 8)
	"*one page is excellent*", "*compact resource*" and "*give answers to all patients' questions in just one sheet*".
	Well presented (n = 19)
	"*well presented*", "*well set out*", "*the layout was similar to other drug information*"
	*General comments about appearance (n = 5)*
	"*clever color coding*", "*written in style doctors understand*", "*handy summarised for quick reference*", "*durable*".
	Content (n = 36)
	"*concise*", "*succinct*," "*comprehensive*", "*the right amount of information*", "*right amount and type of information*", "*evidence based*", "*objective, not overstated*", "*informative*", "*suitable material*", "*excellent accurate information for doctors*".
	Usefulness/helpful (n = 7)
	"*useful*", "*helpful*", "*enjoyed having information both for my own knowledge and for the patients, would like more fact sheets*", "*increased my knowledge*", "*I used the information sheet to write information for patients to take home and decide if they want to use CM*", "*I would love more fact sheets*", "*need more of these resources that GPs can use quickly to gain good sound knowledge about herbs*".

**Short-comings**	**Themes and comments**
	Computerised version (n = 10)
	"*computer database would be best such as in medical director that was continually updated*", "*a cd or computer format*", "*prefer computer stored version*", "*include in MIMs rather than fact sheets*", "*best if available on medical director clinical software to avoid stacking up paper/clinical guidelines*".
	Patient version (n = 3)
	"*not suitable for patients*", "*too technical for patients*", "*patient leaflet e.g*. *tear off tool would be very useful with same information less technical terms*".
	Too much information (n = 4)
	"*slightly too large*", "*size, rapid accessibility during consultation*", "*sheets very busy, a lot of info but better on one page then several*", "*too much information, but I don't know how you could cut it down*".
	Not enough fact sheets- want more on other CMs (n = 5)
	"*not enough of them*", "*not enough medicines covered*", "*more of them*", "*more fact sheet on other commonly used herbs, few sheets isn't enough*".

## Discussion

The series of 4 herbal medicine fact sheets was developed as a result of the needs assessment survey of Qld GPs [7]. Ninety two GPs completed the 4 week evaluation and eighty six (93%) of them used the fact sheets in their clinical practice and the majority (73%) perceived that they were a useful resource.

There are limitations with regards to the generalisability of the sample. The resource was trialled with 100 self-identified GPs in Qld who had expressed interest to evaluate a CMs information resource once it was developed. It is likely that the results are biased towards a more positive outcome, which is of note when generalising the findings from this study. The respondents were over-representative of female and younger GPs compared to the GP population in Australia and Qld. This is consistent with the population studies that have shown that females and younger age groups are more interested in CM than males and older age groups [1,2].

The post-intervention questionnaire was a self-reported instrument which has limitations associated with self-reporting and recall bias. It should also be noted that the attitudinal statements are worded with positive intent and may have attracted biased responses.

Another limitation of this study was that a pre-intervention survey to assess individual changes in GPs' knowledge, attitude, and practice was not conducted prior to implementing the fact sheets, mostly due to limited resources and time. Without a baseline survey, changes in knowledge, attitude and practice could not be calculated [24]. Thus the post-intervention survey could only assess GPs' perceived attitude, knowledge, and practice after having the fact sheets in practice. It is worth noting that the cohort as a whole had identified in the needs assessment [7] that they lacked knowledge of herbal medicine and the primary outcome of the evaluation was to assess the value of the fact sheets in clinical practice.

### GPs' use of the fact sheets in their clinical practice

Over 90% of GPs used the herbal medicine fact sheets at least once, with a third of GPs having used the fact sheets regularly throughout the 4 weeks. The St Johns wort fact sheet was used the most followed by the valerian. This is not unexpected considering that a higher percentage of GPs indicated that they see patients with depression and insomnia for which St Johns wort and valerian are used. The most common reason for GPs using the fact sheets was seeing more patients with the right condition for that herb, or belief that the herb is effective and they perceived the evidence for the specific herb to be strong. This finding verifies that if a GP perceives a particular CMs as useful or beneficial they are more inclined to want to learn more about it, and hence more likely to use available information resources to increase their knowledge.

GPs who used the fact sheets because they believe the herb is effective or the evidence is strong for that herb were more likely to have used the fact sheets for discussing the herb as an option, recommending or prescribing the herb to patients and providing patients with a copy of the fact sheets. These findings reinforce the relationship between GPs' belief of CMs and practice of CMs. Similarly other studies have found that belief in efficacy or legitimacy of selected CM modalities was associated with high frequency of GPs' recommendation of the modality to patients [10,25].

GPs mainly used the fact sheets to increase their own knowledge, answer patient's questions and advise patients. Some respondents used the fact sheets to recommend or prescribe the herbs to patients, and nearly half of the respondents showed the fact sheets to patients, with some GPs even making a copy of the fact sheets for patients. These findings indicate that the fact sheets were not only used successfully by GPs as a learning tool to increase their own knowledge but it also gave GPs the information they required to communicate with patients and to recommend and prescribe the herbs. Furthermore, it confirmed the feasibility of using the fact sheets during consultations to answer patients' questions and advise them about the herbal medicines.

### GPs' assessment of the herbal medicine fact sheets

Nearly all responding GPs perceived that overall the herbal medicine fact sheets contain good quality information. They perceived the content of the fact sheets to be appropriate, sufficient, well presented, easy to comprehend and to use in clinical practice. About 90% of GPs perceived that the fact sheets were useful in assisting them to communicate information about herbal medicines to their patients. This was partly confirmed by other items which showed that 30-40% of GPs indicated that they perceived since having the fact sheets they question patients about their herbal medicine use, and discuss and recommend one or more of the herbal medicines more often than they usually would.

GPs mentioned some shortcomings and ways to improve the fact sheets. Overall, GPs perceived that a computerised version of the fact sheets linked to the medical director which can be continually updated, should be provided in addition to the paper-based fact sheets. This suggestion seems both sensible and feasible. Other studies have shown that a computer-based intervention can increase change in behaviour [26,27]. This could perhaps initially be done for the most commonly used herbal medicines in Australia and eventually all herbal medicines. Other GPs thought that a patient version of the fact sheet with less technical terms would be beneficial, as many of them had photocopied the fact sheet for their patient or written notes from the fact sheet for their patients to take home. This is a valid comment as patient information sheets are often provided for other areas of medicine such as sexually transmitted or communicable diseases, vaccinations, and antibiotics. Another shortcoming was that 4 fact sheets on 4 herbal medicines are not enough for clinical practice and there were suggestions that more fact sheets on other commonly used herbs should be provided.

### Perceived impact on knowledge, attitude, and practice

This study was limited to a post-intervention design which measured GPs' perceived impact of the resource on their attitudes, knowledge, and clinical practice of CMs. It should be made clear that this evaluation offers a snapshot of the value and potential benefits of the resource and is by no means a clear indication of the actual impact of the resource on GPs' attitudes, knowledge, and clinical practice of CMs. Focus group discussions or interviews would have been valuable to further explore the attitudes reported and also reasons for behavioural and attitudinal change.

The herbal medicine fact sheets had a positive impact on GPs' attitude, knowledge and practice of CMs. More than half of responding GPs perceived that prior to receiving the fact sheets they felt they did not have adequate knowledge to discuss those herbal medicine options with their patients. Previous studies [28-31] have also reported that the majority of GPs acknowledge that they have an inadequate level of knowledge to advise patients about CMs.

Approximately 90% of respondents believed that overall the fact sheets increased their knowledge of herbal medicines, and provided them with adequate knowledge to be able to communicate with patients about those specific herbs. This finding shows that the fact sheets did contain appropriate information to facilitate GPs' communication with patients about those herbal medicines. The majority of GPs thought that the fact sheets reassured them that the information they were conveying to patients was correct. This could indicate that sometimes GPs are attempting to discuss CMs with their patients on an uninformed basis and feel somewhat uncertain or not confident about the information they are providing to patients.

A high percentage of GPs (90%) believed that if they had adequate resources on CMs, like the herbal medicine fact sheets, then they would communicate more to their patients about CMs. This confirms that by having an information resource, which provides a basic understanding of different CMs facilitates GPs' confident communication with their patients about CMs [32]. A third of responding GPs believed that they questioned their patients about their herbal medicine use, and discussed herbal medicine options with their patients more often than they usually would have. Almost half of the GPs perceived that they recommended one or more of the herbal medicines mentioned in the fact sheets to the patient with the right conditions more often than they usually would have recommended them. These figures are not surprising as it would be expected that having a herbal medicine information resource on a doctor's desk would prompt questioning of patients, thereby increasing knowledge and providing more confidence to recommend herbal medicines to patients. Several previous studies [10,28,33,34] have confirmed that GPs' knowledge of CMs was associated with GPs' attitudes, practice and referral of CMs, and that if a GP had knowledge of CMs then they would have a more positive attitude and be more willing to practice CMs. Even GPs that have familiarisation with CMs in their medical degree appear to have more positive attitudes towards a patient's interest in discussing CMs [35]. Thus, it is expected that since using the fact sheets, respondents' knowledge of those herbal medicines increased and they became more positive about CMs and were more confident in discussing them. This study also found a positive association between GPs' perceived knowledge of CMs and GPs' perceived practice of CMs. GPs who believed that prior to receiving the herbal medicine fact sheets they felt they did not have adequate knowledge to discuss herbal medicines were more likely to agree that while having the fact sheets, they recommended one or more of the herbal medicines mentioned in the fact sheets to the patients with the right conditions more often than they usually would have. These findings confirm that familiarisation with CMs does impact GPs' practice. Other studies [10,28,33,34] have also demonstrated a positive relationship between GPs' increase in knowledge of CMs and increase in their practice of CMs.

Providing GPs with an opportunity to increase their evidence-based knowledge in their everyday practice by means of information resources on different CMs, like the herbal medicine fact sheets, is likely to increase their knowledge and confidence to discuss CMs with their patients. Suter et al (2004) [30] found a positive relationship between GPs' knowledge about specific CM modalities and their comfort level in discussing CM with their patients. Likewise, Corbin-Winslow and Shapiro (2002) [10] found a linear relationship between increasing comfort levels in discussing CM use with increasing propensity to ask patients about their use of CM modalities. This is logical as GPs feel apprehensive about discussing a topic with patients that they know little or nothing about. Their level of knowledge does not allow them to respond to patients' questions or provide any useful advice, therefore they avoid communication [10]. However, if GPs had adequate resources to increase their knowledge they would be more qualified to evaluate the usefulness of particular CMs modalities objectively and thus be more likely to discuss them with patients.

There was also a positive relationship between GPs' perceived attitude about CMs and their practice of CMs. GPs perceived that since having the fact sheets they discussed and recommended CMs more often than they did prior to the use of the fact sheets. Verhoef and Sutherland (1995) also reported that GPs that had a positive attitude about involvement with CM were more likely to practice CM and refer for CM [36].

It is important to note that the provision of evidence-based information does not imply that it will be translated into practice even when a GP has indicated satisfaction with the intervention or when their knowledge has increased [37-39]. The available literature on the use of printed materials in changing GPs' practices have mostly reported that the dissemination of printed educational materials alone has a small impact on GPs' practice [40-42]. The dissemination of information alone may increase awareness and predispose to change but is not usually sufficient to bring about actual behavioural change in the absence of an active implementation strategy appropriate to the setting concerned [37,43,44]. Another important fact that has been reiterated in literature is that when the goal of an intervention is to change clinical practice, it is necessary to implement a multifaceted intervention incorporating two or more education strategies [42,45-48]. It has been suggested that changing behaviour may be an incremental process requiring a number of exposures to new information before the change occurs [49]. One episode or one series of fact sheets for a new concept may merely raise awareness without inducing change [49]. However, repeated exposures may lead to a "pre-contemplative" GP becoming ambivalent and eventually deciding to implement new practice behaviours [49].

Studies [42,46-48,50] have reported that other educational techniques such as academic detailing and the use of local opinion leaders were the most effective techniques in changing physician performance. Physician reminder systems were also effective, audit and feedback techniques were marginally effective, and conferences and printed materials were the least effective. The passive dissemination of information through the use of mailings, printed materials, lectures, and conferences, although less effective, are substantially less expensive to implement [42,47]. Therefore they are commonly used as a primary method to alter GP behaviour.

Behavioural change is a highly complex process due to the interaction between psychological, cultural, environmental components of the focus behaviour. Given that clinical practice is a form of human behaviour, theories that have proved useful in other similar settings may provide a basis for developing a scientific rationale for the choice of the interventions [47]. Since a number of things influence patterns of practice behaviour for physicians, researchers have been unable to formulate a theory of physician behaviour change. However, psychologists, sociologists and educators have offered several health behaviour change theories that apply to efforts to improve physician performance [51,52]. For example, behavioural/affective theories [53] including the social cognitive theory [54], planned behaviour theory [55-57] and the health belief model suggest that individual's health behaviour change is governed by their goals and perceptions. That is, the more positive people's attitudes, subjective norms and perceived behavioural control, the stronger their intentions to perform the behaviour. Further, the stronger people's intentions, the greater likelihood of their performing the behaviour [58,59]. Walker and colleagues (2001) are exploring the applicability of behavioural theories to professional behaviour [60]. Considering that the GPs in this study had self-identified themselves to trial the CMs' intervention perhaps indicates that they were motivated and had the intention to learn about CM and to bring about change in their practice. Therefore these GPs would be more inclined to use the information in the fact sheet in their clinical practice to bring about behaviour change.

There has long been a clamour for an understanding of what works and what does not work to improve physicians' performance and clinical practice [61-63]. There is an imperfect evidence base as to which dissemination and implementation strategies are likely to be efficient under varying circumstances [47]. However, some attempts have been made to identify what types of educational intervention and the characteristic of interventions that are ideal for changing GPs' behaviour. Smith (1997) concluded that the ideal information source for doctors will need to be directly relevant, contain valid information and be accessed with a minimal amount of work. Such sources include colleagues, drug reference books, journals which contain evidence-based information, regularly updated textbooks, systematic journal reviews and a portable and comprehensive summary of systematic reviews [64]. The literature states that the effectiveness of educational interventions aimed at changing provider behaviour may be influenced by at least 4 factors [40]: (1) characteristics of the intervention, (2) characteristics of the provider, (3) characteristics of the behaviour that the intervention is trying to change and (4) characteristics of the organisation and context. Research on which characteristics of printed educational materials influence clinical practice has been limited [65,66] but some suggestions have been made [65-68]: source (credibility of information), format (e.g. appearance, length), mode of delivery (e.g. mass mailing, direct mailing), frequency/timing of intervention, content of information (e.g., topic, evidence-based). These important characteristics of printed educational materials were all considered in developing the CMs fact sheets of this study to maximise their usefulness and impact on GPs' knowledge, attitude and clinical practice.

## Conclusions

The findings of this study indicate that the production and dissemination of CMs information resources such as the evidence-based herbal medicine fact sheets produced in this study are feasible, worthwhile and have the potential to change and enhance clinical practice, particularly the relationship between doctor and patient regarding CMs. In general, the herbal medicine fact sheets were well accepted and utilised by Qld GPs. The post-intervention evaluation confirmed that such a resource has the potential to increase GPs' knowledge of, and attitudes towards CMs thereby facilitating positive changes in clinical practice. Increasing GPs' knowledge of CMs and improving their communication about CMs could have a substantial impact on the health and well-being of patients. Further educational interventions on CMs need to be provided to GPs on a regular basis to increase their knowledge about CMs so they can confidently communicate with their patients on an informed-basis. Similar to other Australian general practice education programs, future interventions on CMs need to be evaluated as part of a quality cycle to ensure that they are appropriately targeting the needs of general practice and their patients.

## Competing interests

The authors declare that they have no competing interests.

## Authors' contributions

All mentioned authors made substantial contribution to this research and manuscript: TJ carried out all components of the research including development of research protocol, data collection and analysis, and drafted the manuscript. SPM participated in the design of the study, provided important intellectual content and was involved in the drafting of the manuscript. PO participated in the design of the study, provided assistance in the statistical analysis, and was involved in the drafting of the manuscript. HE participated in the design of the study, provided important intellectual content and provided input into the final draft of the manuscript. All authors read and approved the final manuscript.

## Pre-publication history

The pre-publication history for this paper can be accessed here:

http://www.biomedcentral.com/1472-6882/11/77/prepub

## Supplementary Material

Additional file 1 **Herbal medicines fact sheets**. The four herbal medicines fact sheets developed, implemented, and evaluated in this study. Click here for file
