# Supplementary material for: Responding to GPs' information resource needs: implementation and evaluation of a complementary medicines information resource in Queensland general practice
Source: BMC Complement Altern Med. 2011 Sep 20;11:77. doi: 10.1186/1472-6882-11-77 (PMC3190343; doi:10.1186/1472-6882-11-77)
Supplement: Additional file 1 — Herbal medicines fact sheets. The four herbal medicines fact sheets developed, implemented, and evaluated in this study. [file 1472-6882-11-77-S1.PDF]

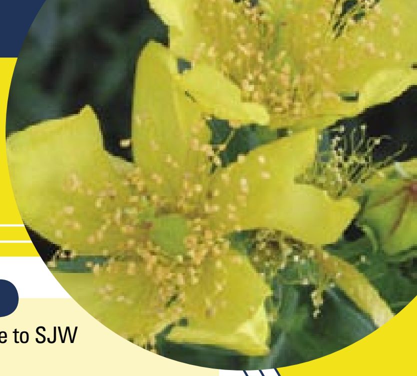

St John's Wort (Hypericum)

OVERVIEW

St John's wort (SJW) is an effective antidepressant. Be aware that it can interact with other drugs.

CLINICAL USES

Primary Use

- **Depression, mild to moderate.** There is convincing evidence of the benefits of SJW for mild to moderate depression. Note SJW should not be used in the treatment of major (severe) depression.

Other Potential Uses

There are studies on the benefits of SJW for some of the following conditions, however, the evidence is currently not at the highest level.

- Healing wounds, first-degree burns, muscle pain (topical preparation for external)
- Anxiety
- Seasonal Affective Disorder (SAD)
- Obsessive-Compulsive Disorder (OCD)
- Menopause
- Fatigue
- Pediatric nocturnal incontinence
- Premenstrual Syndrome (PMS)

MAJOR CHEMICAL COMPONENTS

Constituents include hyperforin and adhyperforin (phloroglucinols), hypericin and pseudohypericin (napthodianthrones), flavonoids, xanthones, oligomeric procyanidines and amino acids.

PHARMACOLOGICAL ACTIONS

SJW inhibits serotonin, noradrenaline, and dopamine; the most potent effect appears to be on gamma-aminobutyric $acid_A$ ($GABA_A$) and $GABA_B$ receptors in vitro. SJW has weak monoamine oxidase (MAO) inhibiting effects. SJW effects on serotonin may be primarily responsible for its antidepressant activity.

DOSAGE and ADMINISTRATION

The onset of response to SJW is similar to that for conventional antidepressants, requiring 2-4 weeks. To prevent relapse, antidepressants should be continued at full therapeutic doses for at least 6 months after remission. After discontinuing a previous antidepressant, allow 2 weeks before starting SJW, and taper the dosage.

- **For depression:** standardised extract (0.3% hypercin and/or 2-5% hyperforin) 300- 900 mg daily in divided doses

ADVERSE EFFECTS

In published studies, SJW has generally been well tolerated at the recommended doses for up to 1-3 months.

Most common adverse effects include:

- Gastrointestinal symptoms (including abdominal pain, bloating, constipation, nausea, and vomiting)
- Skin reactions
- Fatigue/sedation
- Restlessness or anxiety
- Dizziness
- Headache
- Dry mouth

Rare adverse effects include:

- Mania
- Serotonin syndrome (characterised by rigidity, hyperthermia, delerium, confusion, autonomic instability and coma)
- Withdrawal
- Break through bleeding (birth control pill)
- Elevated blood pressure
- Sexual dysfunction (anorgasmia)
- Photosensitization (in fair-skinned patients taking excessive dosages1,800 mg/day)

CONTRAINDICATIONS

- Avoid in patients who are hypersensitive to SJW or to any of its constituents
- Avoid in patients with HIV/AIDS taking protease inhibitors
- Avoid in transplant recipients on cyclosporin
- Use cautiously in patients taking medication metabolised by cytochrome P450, photosensitising drugs, warfarin, monoamine oxidase inhibitors or selective serotonin reuptake inhibitors (SSRI), digoxin, and oral contraception
- Use cautiously in persons with a history of mania, hypomania
- Pregnancy and lactation (safety not established clinically)
- Relative contraindication in children under the age of 18 years (insufficient available evidence)

DRUG INTERACTIONS

Drug-herb interactions have been classified as **confirmed** or **potential** interactions. Confirmed interactions are those validated by clinical trial data or from case reports where causality has been clearly demonstrated. Potential interactions have not been confirmed and are based on theoretical considerations, poor quality case reports or in vitro studies.

Confirmed interactions

- **Drugs metabolised via cytochrome P4503A4-** Concurrent use of drugs metabolised via the CYP4503A4 liver enzyme system may result in decreased plasma levels of pharmacologic agents, due to induction or inhibition of enzymes by SJW

  Examples of medications that may be affected by SJW in this manner include:

  ➔ Cyclosporine, irinotecan, tacrolimus, midazolam, indinavir, nifedipine, birth control pills, simvastatin, theophylline, tricyclic antidepressants, warfarin, or HIV drugs such as non-nucleoside reverse transcriptase inhibitors (NNRTIs) or protease inhibitors (PIs)
- **Selective serotonin Reuptake Inhibitors (SSRI)-** concomitant SJW may lead to increased adverse effects typically associated with SSRI antidepressants (such as fluoxetine or sertraline), including serotonin syndrome
- **Digoxin-** Concurrent use may decrease blood levels of digoxin

Potential interactions

- **Monoamine Oxidase Inhibitors** (MAOI)
- **Tricyclic antidepressants**
- **HMG CoA reductase inhibitors** (Statins)
- **5HT1 receptor agonists** (Triptans)
- **Photosensitising products** (such as some antibiotics, and oral contraceptives)
- **Anesthetic drugs**
- **Opioids**
- **Antineoplastic drugs**
- **Carbamazepine** (CYP 3A4)
- **Loperamide**
- **Midazolam**
- **Nifedipine**
- **Theophylline**
- **Alcohol**
- **Thyroid agents**

OTHER

Latin name: Hypericum perforatum L.
Family: Clusiaceae
Parts used: Flowering tops (leaves, unopened buds, and flowers)
Description: Herbaceous plant with yellow flowers; petals and leaves of SJW have numerous, punctate glands.
Traditional uses: SJW has been used to treat respiratory inflammations, wounds, burns, neuralgia, diuretic, sciatica, nervous disorders, insomnia, anxiety, dyspepsia, menopausal symptoms, migraines, and premenstrual syndrome.

The University Of Queensland

Australian Centre for Complementary Medicine
EDUCATION & RESEARCH

Clinical Trials

Most trials have found SJW effective in treating mild to moderate depression.

- **Linde and Mulrow (1996)-** A meta-analysis of 23 randomised trials of SJW in 1,757 patients with mild-to-moderate depression. Twenty trials were double-blind in design, and 15 were placebo controlled. Results of pooled data from 13 placebo comparison trials revealed a 55.1% response to SJW compared to 22.3% placebo response. Trials were of limited duration (2-8 weeks, with one 12 week trial). The SJW daily dose varied between 300-1000 mg standardised extract with a hypericin daily dose from 0.4 to 2.7mg.
- **Linde and Mulrow (2000)-** Linde and Mulrow followed up with a Cochrane review, in addition to the trials reported in the 1996 meta-analysis, four studies were added. A meta-analysis of 27 trials, including 16 randomized trials, 8 equivalence/ comparison trials, and 3 studies of combination products, with a total of 2291 patients, showed SJW was an effective treatment in mild to moderate depression. Ten studies compared SJW extract with standard reference medications and found it to be equivalent to the tricyclic antidepressants maprotiline, imipramine and amitriptyline, and the benzodiazepines diazepam and bromazepam. Linde and Mulrow concluded that SJW was significantly superior to placebo, and effective comparably with standard antidepressant drugs.
- **Kim et al (1999)-** A meta-analysis of 6 randomised, double-blind controlled trials, including 651 patients. Two studies compared SJW to placebo and four compared it to tricyclic antidepressants. The study concluded that SJW was 1.5 times more likely than placebo to be effective, and was equivalent to short-term, low-dose tricyclic antidepressant treatment of mild-moderate depression.
- **Stevinson and Ernst (1999)-** A review of a further 6 randomised clinical trials which did not overlap with the previous meta-analysis confirmed that SJW is efficacious in treating mild to moderate depression.
- **Gaster and Holroyd (2000)-** A systematic review of SJW monotherapy in depression involved 8 English-language, randomised, controlled, double-blind trials. SJW demonstrated a 23% to 55% absolute increase in response rate higher than placebo, yet 6% to 18% less than tricyclic antidepressants. The results of this review were in general agreement with the Linde and Mulrow meta-analysis.
- **Schrader (2000) and Harrer et al (1999)-** Two recent double-blind placebo-controlled RCTs compared SJW to fluoxtine (Prozac). One tested SJW (Ze 117; 250 mg twice daily) against fluoxetine (20 mg) for 6 weeks in 240 patients with mild to moderate depression (Schrader 2000); the other tested a dry extract (LoHyp-57, 400 mg twice daily) against sertraline (10 mg twice daily) x 6 weeks in 149 participants (129 female). Both trials found the treatments equivalent.
- **Philipp et al (1999) and Woelk (2000)-** Two other recent trials found SJW equivalent to imipramine. One compared SJW extract (350 mg three times a day) to imipramine (100 mg/day in divided doses) for 8 weeks in 263 patients (197 women) with moderate depression; the other tested Ze 117 (250 mg twice daily x 6 weeks) against imipramine (75 mg twice daily) in 324 patients with mild to moderate depression. In both trials, treatments were equivalent.
- **Whiskey et al (2001)-** The most recent meta-analysis, analysed 22 randomised controlled trials, and concluded that SJW is significantly more effective than placebo and as effective as standard pharmaceutical antidepressant drugs in the treatment of depression.

SUMMARY

There is a substantive amount of data that suggests that SJW is safe, well tolerated, and more effective than placebo in treatment of mild to moderate depression. Some studies have shown SJW to be at least as effective as standardised antidepressant drugs in treating depression. However, SJW has not been found effective for treatment of major (severe) depression.

References

Linde K, Ramirez G, Mulrow CD, et al. St John's wort for depression--an overview and meta-analysis of randomised clinical trials. *BMJ 1996;313(7052):253-258.*

Linde K, Mulrow CD. St. John's wort for depression. *Cochrane Database Syst Rev 2000;(2):CD000448.*

Kim HL, Streltzer J, Goebert D. St. John's wort for depression: a meta-analysis of well-defined clinical trials. *J Nerv Ment Dis 1999;187(9):532-539.*

Stevinson C, Ernst E. Hypericum for depression. An update of the clinical evidence. *Eur Neuropsychopharmacol 1999;9(6):501-505.*

Gaster B, Holroyd J. St John's wort for depression: a systematic review. *Arch Intern Med 2000;160(2):152-156.*

Schrader E. Equivalence of St John's wort extract (Ze 117) and fluoxetine: a randomised, controlled study in mild-moderate depression. *Int Clin Psychopharmacol 2000;15:61-68.*

Harrer G, Schmidt U, Kuhn U et al. Comparison of equivalence between the St. John's wort extract LoHyp-57 and fluoxetine. *Arzneim-Forsch/Drug Res 1999;49:289-296.*

Philipp M, Kohnen R, Hiller KO. Hypericum extract versus imipramine or placebo in patients with moderate depression: randomised multicentre study of treatment for eight weeks. *BMJ 1999;319:1534-1538.*

Whiskey E, Werneke U, Taylor D. A systematic review and meta-analysis of Hypericum perforatum in depression: a comprehensive clinical review. *Int Clin Pyschopharmacol 2001;16(5):239-252.*

This Herbal Medicine Fact Sheet is one of a series of fact sheets that have been produced by the Australian Centre for Complementary Medicine Education and Research (ACCMER) and the School of Population Health of the University of Queensland for GPs to use in their clinical practice. These fact sheets are currently being piloted and evaluated and if successful will be widely distributed to GPs in the future.

Ginkgo Biloba

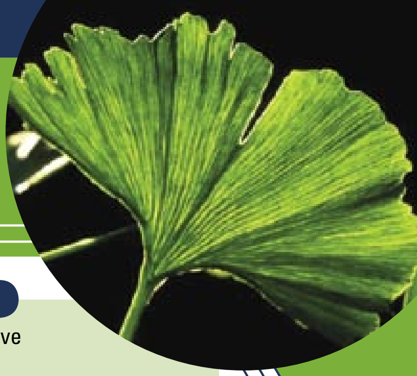

OVERVIEW

Ginkgo is useful in treating dementia, age-related memory problems, and intermittent claudication. In rare cases it can increase risk of bleeding.

CLINICAL USES

Primary Uses

There is convincing evidence of the benefits of ginkgo for the following conditions.

- **Dementia** (Alzheimer's/multi-infarct dementia)
- **Cerebral insufficiency** (impaired concentration, confusion, decreased physical performance, fatigue, headache, dizziness, depression, and anxiety)
- **Intermittent claudication**- peripheral vascular disease including poor circulation to the lower legs

Other Potential Uses

There are studies on the benefits of ginkgo for some of the following conditions, however, the evidence is currently not at the highest level.

- Memory enhancement
- Ringing in ears (tinnitus)
- Dizziness or whirling sensation (vertigo)
- Sexual dysfunction associated with use of SSRI drugs
- Control of acute symptoms of altitude sickness and vascular reactivity to cold exposure
- Protective action in hypoxia
- Acute deafness related to the cochlea

MAJOR CHEMICAL COMPONENTS

Constituents include flavonoid glycosides and terpenoids (ginkogolides, bilobalide) which are considered to be ginkgo's primary active components.

PHARMACOLOGICAL ACTIONS

Antioxidant/anti-inflammatory effects - Flavonoids serve as free-radical scavengers, and have been shown to reduce oxidative stress in human models. Ginkgolides inhibit receptor binding of platelet activating factor, which may mediate beneficial clinical effects.

Neurotransmitter effects - Neuroprotective properties have been attributed to inhibition of age-related decline of adrenergic and cholinergic receptors. Ginkgo also increases serotonin levels, increases muscarinic binding sites, and increases serum levels of acetylcholine and norepinephrine.

Vascular effects - Ginkgo has vasodilatory effects, which have been attributed to stimulation of endothelium-derived relating factor and prostacyclin release. Ginkgo also significantly increases blood capillary flow and decrease erythrocyte aggregation.

DOSAGE and ADMINISTRATION

Initial results often take 4-6 weeks, but should continue to accumulate beyond that time period.

- **For dementia and memory impairment:** 120 to 240 mg of a 40:1 standardised extract per day in divided doses (standardised to 24-25% ginkgo flavone glycosides)
- **For peripheral arterial disease, tinnitus, and vertigo:** 120-160 mg per day in divided doses

ADVERSE EFFECTS

Ginkgo is usually well tolerated at the recommended dose for up to six months and adverse effects are uncommon.

Most common adverse effects include:

- Mild gastrointestinal disturbance
- Headaches, dizziness, restlessness

Rare adverse effects include:

- Bleeding- subarachnoid hemorrhage, subdural hematoma, and postoperative bleeding
- Seizures
- Decrease systolic and diastolic blood pressure and palpitations
- Increase plasma insulin concentrations in healthy individuals and decrease these concentrations in type 2 diabetes

CONTRAINDICATIONS

- Avoid in patients who are hypersensitive to ginkgo biloba preparations
- Avoid in patients at risk of bleeding, taking anti-coagulants, or with clotting disorders
- Discontinue use 2-3 weeks prior to some surgical and dental procedures due to increased risk of bleeding
- Use cautiously in patients with seizures
- Use cautiously in children
- Avoid in couples who are trying to conceive, based on theoretical reduction of fertility
- Pregnant or lactating women (lack of sufficient data)

DRUG INTERACTIONS

Drug-herb interactions have been classified as **confirmed** or **potential** interactions. Confirmed interactions are those validated by clinical trial data or from case reports where causality has been clearly demonstrated. Potential interactions have not been confirmed and are based on theoretical considerations, poor quality case reports or in vitro studies.

Confirmed interactions

None known

Potential interactions

- **Anticoagulants, antiplatelet drugs, NSAIDS, Pentoxifylline-** affects platelet activation factor and increase risk of bleeding when taken with the following: aspirin, dipyridamole, heparin, warfarin, carbamazepine, clopidogrel, ticlopidine
- **Anticonvulsants-** can decrease the effectiveness of anticonvulsants such as carbamazepine and sodium valproate in patients with coexisting symptoms of cerebral insufficiency
- **Antihypertensive medications-** may have additive effects when used with these drugs
- **5-fluorouracil, cyclosporine-** 5-fluorouracil induces adverse effects and cyclosporine nephrotoxicity may be alleviated by ginkgo
- **Papaverine-** Potentiate the actions of other drugs used in management of vascular erectile dysfunction
- **Thiazide diuretics-** Increase blood pressure when taken with thiazide diuretics
- **Trazodone-** may increase the sedative effects of the drug trazodone
- **Hypoglycaemic oral agents, insulin-** May cause elevated blood glucose levels by facilitating increased clearance of both insulin and oral hypoglycaemic agents
- **Selective Serotonin Reuptake Inhibitors (SSRI's)-** ginkgo may act additively with SSRI's, with a risk of causing serotonin sydrome
- **Other drug interactions-** acetylcholinestrase Inhibitor Drugs (donepezil and tacrine), antipsychotic drugs, colchicine, Monoamine Oxidase Inhibitors (MAOI's)

OTHER

Other common names: Maidenhair tree
Latin name: Ginkgo biloba
Family: Ginkgoaceae
Parts used: Leaves
Description: An ancient tree, existed 100 million years ago. A common urban planting, it has distinctive fan-shaped leaves.
Traditional uses: The leaves were not used in traditional medicine.

The University Of Queensland

Australian Centre for Complementary Medicine
EDUCATION & RESEARCH

Clinical Trials

Most trials have found ginkgo effective in treating dementia, Alzheimer's disease, cerebral insufficiency, and intermittent claudication

DEMENTIA / MEMORY IMPAIRMENT

- **Birks et al (2002)-** A 2002 Cochrane Systematic Review concluded that ginkgo biloba produces benefits in people with acquired cognitive impairment, including dementia compared with placebo in a dose less than 200mg/day in less than 12 weeks treatment. Activities of Daily Living (ADL) and measures of mood and emotional function show benefit for ginkgo also. There were no significant differences between ginkgo and placebo in the proportion of participants experiencing adverse events. The authors concluded that ginkgo biloba appears to be safe in use with no excess side effects compared to placebo. Overall, there is promising evidence of improvement in cognition and function associated with ginkgo.
- **Ernst and Pittler (1999)-** A systematic review of nine randomised, double-blind, and placebo-controlled studies also concluded that ginkgo is more effective than placebo for dementia.
- **Oken et al (1998)-** A meta-analysis identified more than 50 trials on ginkgo leaf extract and dementia and Alzheimer's, however, only 4 studies with a total of 424 participants met inclusion criteria. The authors concluded that there is a small but significant effect of 3 to 6 months of treatment with 120- 240 mg ginkgo extract on cognition in Alzheimer's disease without causing adverse effects.
- **Le Bars (1997)-** A well designed randomized, double-blind, placebo controlled, parallel-group, multicentre study in 309 patients with mild-to-severe dementia. Subjects received ginkgo (120mg daily) or placebo during a 52-week trial period. Overall, the ginkgo group scored better than placebo on the Alzheimer's Disease Assessment Scale-Cognitive subscale (ADAS-Cog), and better than placebo on the Geriatric Evaluation by Relative's Rating Instrument (GERRI).
- **Wettstein (2000)-** A review published in 2000 found that acetylcholinesterase inhibitors and ginkgo yielded similar improvements in dementia symptoms on the same validated scale (ADAS-Cog) compared to placebo. The author concluded that ginkgo extract should be considered equally effective as second generation cholinesterase inhibitor drugs (donepezil, rivastigmine, metrifonate) in the treatment of mild to moderate Alzheimer's dementia.
- **Itil et al (1998)-** A small randomised study showed a dose of 240 mg of ginkgo biloba produced typical cognitive activator ECG profiles in more subjects than 40 mg of the drug tacrine.
- **Kleijnen and Knipschild (1992)-** A meta-analysis of 40 controlled trials of ginkgo for "cerebral insufficiency" (memory impairment not dementia). It found that 26/40 trials showed that ginkgo benefited cerebral insufficiency. The authors concluded that ginkgo biloba is an effective treatment, however, only eight studies were considered to be well conducted.

INTERMITTENT CLAUDICATION

- **Pittler and Ernst (2000)-** A meta-analysis of eight randomised, double blind, placebo-controlled clinical trials found that ginkgo significantly increased pain-free walking distance.
- **Kleijnen and Knipschild (1992)-** A review of 15 controlled trials of ginkgo for intermittent claudication found a benefit in all trials; however, only 2 trials were of acceptable quality.
- **Letzel and Schoop (1992)-** Comparative studies of ginkgo to the drug pentoxifylline (the gold standard treatment in Europe for intermittent claudication) suggest that both treatments are similarly effective in increasing the walking distance of patients with claudication. The effect has been described as clinically relevant (Ernst 2002).

SUMMARY

The available evidence suggests that ginkgo extract is an effective treatment of dementia and Alzheimer's disease with very few adverse events. Data from multiple small randomised controlled trials and two meta-analyses suggest that oral ginkgo modestly improves symptoms of intermittent claudication.

References

Birks J, Grimley EV, Van Dongen M. Ginkgo biloba for cognitive impairment and dementia. *Cochrane Database Syst Rev 2002;(4):CD003120.*

Ernst E, Pittler MH. Ginkgo biloba for dementia: a systematic review of double-blind, placebo-controlled trials. *Clin Drug Invest 1999;17:301-308.*

Oken BS, Storzbach DM, Kaye JA. The efficacy of ginkgo biloba on cognitive function in Alzheimer disease. *Arch Neurol 1998;55:1409-1415.*

Le Bars PL, Katz MM, Berman N, et al. A placebo-controlled, double-blind, randomised trial of an extract of ginkgo for dementia. North American EGb Study Group. *JAMA 1997;278(16):1327-1332.*

Wettstein A. Cholinesterase inhibitors and ginkgo extracts—are they comparable in the treatment of dementia? Comparison of published placebo-controlled efficacy studies of at least six months' duration. *Phytomedicine 2000;6(6):393-401.*

Itil TM, Eralp E, Ahmed I, Kunitz A, Itil KZ. The pharmacological effects of Ginkgo biloba, a plant extract, on the brain of dementia patients in comparison with tacrine. *Psychopharmacol Bull 1998;34(3)391-397.*

Kleijnen J, Knipschild P. Ginkgo biloba for cerebral insufficiency. *Br J Clin Pharmacol 1992;34(4):352-358.*

Pittler MH, Ernst E. Ginkgo biloba extract for the treatment of intermittent claudication: a meta-analysis of randomised trials. *Am J Med 2000;108(4):276-281.*

Kleijnen J, Knipschild P. Ginkgo biloba. *Lancet 1992;340:1136-1139.*

Letzel H, Schoop W. Ginkgo biloba extract EGb 761 and pentoxifylline in intermittent claudication. Secondary analysis of the clinical effectiveness. *Vasa 1992;21(4):403-410.*
Ernst E. The risk-benefit profile of commonly used herbal therapies: Ginkgo, St. John's Wort, Ginseng, Echinacea, Saw Palmetto, and Kava. *Ann Intern Med 2002;136(1):42-53.*

This Herbal Medicine Fact Sheet is one of a series of fact sheets that have been produced by the Australian Centre for Complementary Medicine Education and Research (ACCMER) and the School of Population Health of the University of Queensland for GPs to use in their clinical practice. These fact sheets are currently being piloted and evaluated and if successful will be widely distributed to GPs in the future.

Valerian

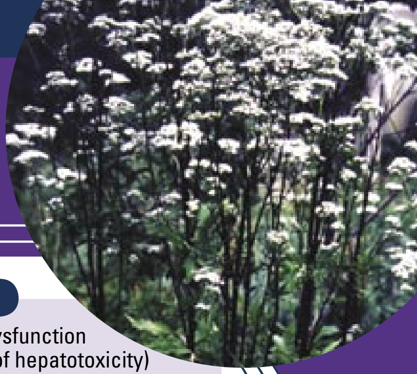

OVERVIEW

Valerian is a benign sedative-hypnotic; clinical trials support its use.

CLINICAL USES

Primary Uses

Preliminary data support the use of valerian for insomnia and anxiety.

- **Insomnia:** Valerian can be used for sleep disorders, insomnia, and restlessness based on nervous disorders
- **Anxiety:** The World Health Organization supported by clinical data, lists uses as a mild sedative and sleep-promoting agent; a milder alternative or possible substitute for stronger synthetic sedatives (e.g., benzodiazepines); and for treatment of nervous excitation and sleep disturbances induced by anxiety

MAJOR CHEMICAL COMPONENTS

The primary active constituents can be divided into four categories:

1. **Valepotriates (iridoids)** - such as valtrate, isovaltrate, acevaltrate, dihydrovaltrate
2. **Volatile oil** - contains valerenic acid, kessanes, valerenal, and valeranone
3. **Amino acids** - arginine, GABA, glutamine, tyrosine
4. **Alkaloids** - Pyridine type; actinidine, chatinine, skyanthine, valerianine, and valerine

PHARMACOLOGICAL ACTIONS

Mechanism has not been definitely established. The pharmacological effects of valerian have primarily been attributed to valepotriates (iridoid esters), volatile oils, monoterpenes, and sesquiterpenes constituents.

Valepotriate constituents are known to have sedative-hypnotic and spasmolytic effects. The sesquiterpenes, valerenic acid and kessyl glycol have been shown to cause sedation in animals. Valerenic acid also appears to inhibit the enzyme system responsible for the central catabolism of GABA, increasing GABA concentrations and decreasing central nervous system activity, resulting in sedation.

DOSAGE and ADMINISTRATION

Composition and purity of valerian vary greatly.

For sleep disorders: 400 to 900 mg of standardised valerian extract (contains 0.8% valerenic acid, 1% to 1.5% valtrates) to be taken 30-60 minutes before bedtime.

ADVERSE EFFECTS

The level of adverse effects reported in clinical trials has not been greater than placebo.

Most common adverse effects include:

- Headache
- Excitability
- Insomnia
- Ataxia and hypothermia
- Dizziness and shakiness
- Although residual morning effects on alertness and concentration appear uncommon, impaired alertness and information processing may sometimes occur. Impairment is dose-dependent and peaks within the first few hours after an oral valerian dose

CONTRAINDICATIONS

- Use cautiously in patients with liver dysfunction (due to an unconfirmed potential risk of hepatotoxicity)
- Should not drive or operate machinery after consuming valerian
- Use cautiously in patients taking other sedative agents due to risk of additive effects
- Pregnancy and lactation (safety not established clinically)
- Relative contraindication in children (insufficient available evidence)

DRUG INTERACTIONS

Drug-herb interactions have been classified as **confirmed** or **potential** interactions. Confirmed interactions are those validated by clinical trial data or from case reports where causality has been clearly demonstrated. Potential interactions have not been confirmed and are based on theoretical considerations, poor quality case reports or in vitro studies.

Confirmed interactions

None Known

Potential interactions

- **Alcohol (ethanol)-** might have an additive sedative effect with alcohol
- **Sedative drugs/CNS depressants and benzodiazepines-** concomitant use of valerian and drugs with sedative properties may cause additive therapeutic and adverse effects
- **Cytochrome P450 3A4 (CYP3 A4) substrates-** valerian may inhibit the cytochrome P450 3A4 (CYP3 A4) enzyme. Theoretically, valerian might increase levels of drugs metabolised by CYP3 A4
- **Selective Serotonin Reuptake Inhibitors (SSRIs)-** may cause mental status changes when taken with antidepressant such as fluoxetine (Prozac)
- **Disulfiram (Antabuse) and metronidazole (Flagyl)-** alcohol content in some valerian extracts may lead to vomiting if used with the drug disulfiram (Antabuse) or metronidazole (Flagyl)
- **Anti-seizure agents-** may interact with anti-seizure drugs
- **Loperamide (Imodium)-** may cause acute delirium
- **Beta Blockers**
- **Narcotics** (such as codeine)
- **Barbiturates** (such as Phenobarbital)
- **Antihistamines** (such as diphenhydramine (Benadryl))

OTHER

Latin name: Valeriana officinalis
Family: Valerianaceae
Parts used: Root, rhizome
Description: Has a white to pale pink, scented flower. It grows in meadows or woods in Europe and North America and is cultivated in Europe, Japan, and Russia. Used medicinally for at least 2,000 years, it was known to Dioscorides and Galen.
Traditional uses: Insomnia, nervousness, stress-related anxiety, migraines, stomach or intestinal cramps, hysteria, exhaustion, attention deficit/hyperactivity disorder, and abdominal, pelvic, or menstrual cramps. It has also been used for flatulence, congestive heart failure, and angina pectoris. Topically, has been used to treat skin sores and acne.

The University Of Queensland
Australian Centre for Complementary Medicine
EDUCATION & RESEARCH

Clinical Trials

Preliminary data supports the effectiveness of valerian for treating insomnia.

INSOMNIA

- **Stevinson and Ernst (2000)-** A well-designed systematic review of randomised, placebo controlled, double-blind clinical trials of valerian in insomnia. Nine trials (from eight studies), published between 1984-1996, met the selection criteria. Of these, six trials examined acute effects of valerian, while three examined cumulative effects. Several of these trials reported positive effects on sleep latency or quality but all had methodological flaws. Pre-bedtime variables (e.g., caffeine, alcohol) were not fully controlled in any study. The authors concluded that the results are promising but inconclusive.
- **Ziegler et al (2002)-** A randomised, controlled, double-blind comparison study, published after the Stevinson and Ernst systematic review. The authors randomised 202 adult outpatients with insomnia of mean duration 3.5 months, to receive either 600mg/day of valerian standardised extract LI 156 or 10mg/day benzodiazepine oxazepam. Following six weeks of therapy, no statistically significant mean differences were detected between groups in sleep quality on the Sleep Questionnaire B (SF-B; CIPS 1996), clinical global impression scale (CGI), or several other validated measures of sleep and fatigue. Treatment was rated as "very good" by 83% of valerian patients, and by 73% of oxazepam patients. Adverse effects were rated as mild or moderate in 28% of valerian patients and in 36% of oxazepam patients. These results are promising, although without a placebo arm to demonstrate improvements in both the valerian and oxazepam arms compared to control, these results cannot be considered definitive.
- **Vobach et al (1996)-** Well-designed, 28-day multi-centre, double-blind, placebo controlled parallel group study of 121 patients with non-organic insomnia according to ICD-10 criteria. The treatment group received a dry ethanolic valerian extract (Seronium) at a dose of 600mg one hour before bedtime. Effectiveness was judged according to the Goetelmeyer sleep questionnaire B, Zerssen well-being scale, and Clinical Global Impression (CGI) scale/global clinical impression scale. Pre-trial evaluation showed no relevant baseline differences. Interim results at 14 days demonstrated statistically insignificant trends in favor of valerian for most measures, although evaluations of therapeutic effectiveness by the physician on CGI scale attained statistical significance ($p<0.05$). At 28 days, statistically significant differences in favour of valerian were seen in all primary outcomes. This study suggests that valerian's effects may be cumulative, with optimal effects occurring with persistent use over a period of at least one month.
- **Dorn et al (2000)-** Well-designed double blind trial (n=75) comparing a dry ethanolic valerian extract (Sedonium, 600mg/day) with benzodiazepine oxazepam (10mg/day). The sample included primarily older female patients who had complained of insomnia for over one year. The primary outcome measure was sleep quality as defined by the SF-B questionnaire. Patients took valerian or oxazepam for 28 days. Both oxazepam and valerian groups showed significant improvements in sleep quality over baseline ($p<0.001$), and the two groups were not statistically different from each other in their efficacy. The effect size was small for both groups (between 0.02 and 0.05), with valerian exhibiting a more favourable adverse event profile. Lack of placebo and small effect sizes overall raise questions about benefits of therapy in either group.
- **Leathwood et al (1982)-** A double-blind, randomised trial using self-rated measures to assess sleep latency and sleep quality in 128 healthy participants. Subjects received either 2 placebo capsules, 2 specially-prepared Hova capsules (containing 200mg valerian and 100mg hops (flores humuli)}, or 2 valerian capsules for one night before bedtime. The medications were administered on two consecutive evenings. A questionnaire was filled out by subjects, reflecting back on their sleep quality after each treatment. Results showed a significant improvement in subjective assessments of sleep latency (decreased time until falling asleep) with valerian monotherapy (37% of patients) and Hova (31%) vs. placebo (23%). The data presented for sleep quality state that 43% of subjects reported improved sleep after taking valerian monotherapy vs. 25% for placebo. Although there were flaws with the methodology, this study does suggest that the effects of valerian may be clinically insignificant in healthy patients with no reported sleep disturbances.

ANXIETY

- **Andreatini et al (2002)-** A randomised, controlled, double-blind trial in 36 outpatients with generalised anxiety disorder, diagnosed by DSM II criteria. Following a two-week washout period, subjects were randomised to receive valerian extract (mean daily dose 81mg), diazepam (mean daily dose 6.5mg), or placebo. No significant difference was observed among the 3 groups at baseline or in the change from baseline on the Hamilton anxiety scale (HAM-A) or in the trait part of the state-trait anxiety inventory (STAI-trait). The 3 groups presented a significant reduction in the total HAM-A scores. Only the diazepam and valerian groups showed a significant reduction in the psychic factor of HAM-A. The diazepam group also presented a significant reduction of the STAI-trait. The authors concluded that the study suggest that valerian may have a potential anxiolytic effect on the psychic symptoms of anxiety, however further studies need to be done with a larger sample.

SUMMARY

Preliminary data from several human trials suggest that valerian improves subjective measures of sleep quality and sleep latency, for up to 4-6 weeks. Better effects have been noted in poor sleepers. Preliminary evidence suggests that ongoing use may be more effective that acute (single-dose) use, with progressive effects over 4 weeks. However, most available studies have been methodologically weak, and in most cases results have not been confirmed using objective sleep pattern data in a sleep laboratory. Valerian has also been proposed as a treatment for anxiety, however further evidence is warranted before a strong recommendation can be made.

References

Stevinson C, Ernst E. Valerian for insomnia: a systematic review of randomised clinical trials. *Sleep Med 2000;1:91-99.*

Ziegler G, Ploch M, Miettinen-Baumann A, et al. Efficacy and tolerability of valerian extract LI 156 compared with oxazepam in the treatment of non-organic insomnia-- a randomised, double-blind, comparative clinical study. *Eur J Med Res 2002;7 (11):480-486.*

Vorbach EU, Darmstadt R, Gortelmeyer, et al. Therapie von insomnien. *Psychopharmakotherapie 1996;3:109-115.*

Dorn M. Efficacy and tolerability of Baldrian versus oxazepam in non-organic and non-psychiatric insomniacs: a randomised, double-blind, clinical, comparative study. *Forsch Komplementarmed Klass Naturheilkd 2000;7(2):79-84.*

Leathwood PD, Chauffard F, Heck E, et al. Aqueous extract of valerian root (Valeriana officinalis L.) improves sleep quality in man. *Pharmacol Biochem Behav 1982;17(1):65-71.*

Andreatini R, Sartori VA, Seabra ML, et al. Effect of valepotriates (valerian extract) in generalised anxiety disorder: a randomised placebo-controlled pilot study. *Phytother Res 2002;16(7):650-654.*

This Herbal Medicine Fact Sheet is one of a series of fact sheets that have been produced by the Australian Centre for Complementary Medicine Education and Research (ACCMER) and the School of Population Health of the University of Queensland for GPs to use in their clinical practice. These fact sheets are currently being piloted and evaluated and if successful will be widely distributed to GPs in the future.

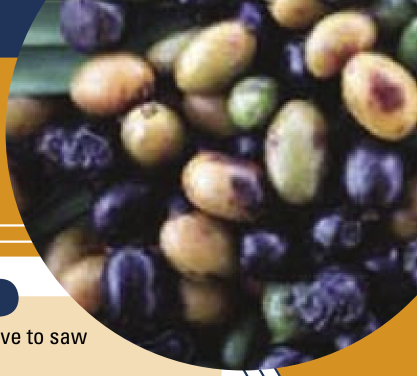

Saw Palmetto

OVERVIEW

Clinical trial evidence supports efficacy of saw palmetto for treating benign prostatic hyperplasia (BPH); prostate-specific antigen (PSA) is not affected. Does not appear to have negative effects on sexual function.

CLINICAL USES

Primary Uses

- **Benign prostatic hypertrophy (BHP), stages I and II.** There is convincing evidence of the benefits of saw palmetto for BHP.

Other Potential Uses

There are studies on the benefits of saw palmetto for some of the following conditions, however, the evidence is currently not at the highest level.

- Genitourinary problems including low sperm count and lack of libido
- Androgenic alopecia (topical)
- Hypotonic neurogenic bladder
- Prostatitis/chronic pelvic pain
- Migraine headaches, inflammations, bronchitis, diabetes, and cancer

MAJOR CHEMICAL COMPONENTS

Constituents include saturated and unsaturated free fatty acids (capric, caprylic, caproic, lauric, palmitic, and oleic acids); free and conjugated sterols; fruits also contain high-molecular weight polysaccharides and flavonoids.

PHARMACOLOGICAL ACTIONS

Multiple mechanisms of action have been proposed.

Hormonal/estrogenic effects- inhibits 5-α-reductase activity on testosterone, thereby preventing the conversion of testosterone to dihydrotestostrone (DHT), and possibly exerting an anti-tumor effect.

Androgen receptor effect- inhibits androgen activity via competition with DHT at the androgen receptor.

Adrenergic effects- anti-α_1-adreonoceptor activity. Anti-proliferative Effects- disrupts intra-cellular nuclear membranes of prostate cells, yielding increased apoptosis.

Anti-inflammatory effects- inhibits lipooxygenase and cyclooxygenase.

DOSAGE and ADMINISTRATION

For BPH: 320mg daily in one or two divided doses (standardized extract usually contains 80-90% sterols and fatty acids (liposterolic content)). Duration of treatment has usually been 3 months (up to 6 months).

Complementary medicine practitioners may recommend other preparations including: 1 or 2 grams of ground, dried berries daily; decoction 0.5 to 1.0 g 3 times a day; or fluid extract of berry pulp 1-2mL 3 times a day.

ADVERSE EFFECTS

Generally well tolerated by most patients for up to 3-5 years.

Most common adverse effects include:

- Gastrointestinal- abdominal discomfort or pain, nausea, vomiting, diarrhoea, and rare reports of duodenal ulcers and cholestatic hepatitis
- Mild headaches
- Dizziness
- Fatigue and muscle pain

Rare adverse effects include:

- Depression
- Sexual dysfunction- decreased libido and impotence
- Cardiovascular- breathlessness and pulmonary emboli, hypertension, tachycardia, angina pectoris, atrial arrhythmia, extra systole, angiopathy, myocardial infarction, and congestive heart failure
- Bleeding- intra-operative, cerebral haemorrhage
- PSA level artificially decreased and may delay diagnosis of prostate cancer

CONTRAINDICATIONS

- Avoid in patients who are hypersensitive to saw palmetto or any of its constituents
- Use cautiously in patients with bleeding disorder
- Use cautiously in patients taking anti-androgens such as finasteride
- Use cautiously in patients with gastrointestinal disorders
- Use cautiously in patients with hypertension
- Avoid the supplement/herb combination products which may contain warfarin and alprazolam
- Avoid in women who are pregnant, lactating, or on HRT or oral contraception

DRUG INTERACTIONS

Drug-herb interactions have been classified as **confirmed** or **potential** interactions. Confirmed interactions are those validated by clinical trial data or from case reports where causality has been clearly demonstrated. Potential interactions have not been confirmed and are based on theoretical considerations, poor quality case reports or in vitro studies.

Confirmed interactions

- **Anti-hypertension agents-** cases of hypertension have been reported in trial of subjects taking saw palmetto

Potential interactions

- **Anti-androgenic drugs-** such as 5-α-reductase inhibitor finastrine; the androgen receptor antagonist bicalutamide, flutamide, nilutamide; or the GnRH antagonists leuprolide, goserelin, and histrelin
- **Androgenic drugs-** testosterone, methyltestosterone, fluoxymesterone, nandrolone decanoate, or stanozolol
- **Estrogen, HRT, Oral contraception-** interferes with these drugs
- **Anticoagulants, anti-platelet agents, NSAIDs-** should be avoided with such agents that increase risk of bleeding

OTHER

Latin name: Serenoa repens or Sabal serrulata
Family: Arecaceae
Parts used: Dried ripe fruit
Description: The most common palm in the United States, is 6-10 ft tall and has fan shaped leaves up to 1 m, and black fruits.
Traditional uses: Used as an expectorant and to treat general debility, and respiratory catarrh. Tonic for both female and male reproductive systems and urinary tracts, nourishing tonic, anti-inflammatory, urinary antiseptic, relaxant, diuretic, cystitis, testicular atrophy, to increase sexual vigor, dysentery, hirsutism.

The University Of Queensland
Australian Centre for Complementary Medicine
Education & Research

Clinical Trials

Numerous controlled trials have found saw palmetto effective in treating benign prostatic hypertrophy (BPH).

- **Wilt et al (2000)-** Well-designed systematic review of 16 trials involving 2939 patients with BPH who had taken saw palmetto extracts for a minimum of 4 weeks. Compared to placebo, saw palmetto was associated with significant improvements in urinary tract symptom scale scores, with an absolute improvement vs. placebo of 28%; a significant improvement in nocturia nightly events; and a significant improvement in self-rated urinary tract symptoms. Compared to finasteride, saw palmetto appeared to yield similar improvements in urinary tract symptoms measured via the International Prostate Symptoms Scale. The evidence suggests improvements in urinary symptoms and flow compared to placebo, and similar effectiveness compared to finasteride with fewer side effects.
- **Wilt et al (2002)-** A more recent systematic review published in 2002 included 3 new trials and produced the same result. Compared to placebo, saw palmetto improves urinary symptom scores, symptoms, and flow measures in men with BPH.
- **Boyle et al (2000)-** A meta-analysis of clinical trial data available for the standardized saw palmetto extract, Perimixon. The authors examined 11 randomized clinical trials (4 placebo controlled, 4 compared to α-blockers or finasteride) and 2 open label trials for an aggregate patient number of 2,859. Pooled data revealed Perimixon (320mg daily) to increase Qmax rates by 2.2mL/s relative to placebo, and to reduce the average number of episodes of nocturia by 0.5 episodes versus placebo. The significant finding of increased urinary flow rate compared to placebo is compelling.
- **Mohanty et al (1999)-** A randomized, double-blind, placebo controlled trial of saw palmetto extract in 75 men with mild-to-moderate symptomic ("grade I-II) BHP. After 8 weeks, the authors reported significant reduction in mean symptom score in the saw palmetto group compared to the placebo group (81% reduction vs. 64% reduction, respectively). In addition, a secondary outcome of "grouped response" (excellent, good, satisfactory) yielded a significant overall superiority of saw palmetto compared to placebo, with response in 76% of the extract group compared to 35% in the placebo group.
- **Gerber et al (2001)-** A randomized, controlled, double-blind trial of saw palmetto extract in 85 men with lower urinary tract symptoms. After a 4-week placebo run-in period, subjects were randomized to either placebo or saw palmetto at a dose of 160mg twice daily for 24 weeks. The authors reported significant decrease in mean IPSS of 4.4 points in the saw palmetto group (16.7 to 12.3) compared to the mean score reduction of 2.2 in the placebo group (15.8 to 13.6). However, there was no significant difference between groups in urinary flow rate.
- **Bauer et al (1999)-** A randomized, double-blind, placebo controlled trial evaluating the effects of saw palmetto in 101 men with BHP. Patients were randomized to receive 160mg twice daily of saw palmetto or placebo for 24 weeks. Statistically significant mean reductions in urinary obstruction (31.8%), and dysuria (38.3%) but not post-void residual volume were observed in the treatment group compared to the placebo group. A significant mean improvement in quality of life was also noted among those ingesting saw palmetto vs. placebo.
- **Carraro et al (1996)-** A trial comparing the effects of saw palmetto and finasteride in the treatment of 1,098 men with BPH diagnosed by digital rectal exam. Patients were randomized to receive either 160mg Permixon twice daily or 5mg finasteride daily (with placebo in the evening) for 26 weeks. In these subjects, the authors reported that 38% decrease in mean symptoms score occurred in both groups, and urinary flow improved in both groups by 36-39% from baseline. Sexual function was unchanged in the Permixon group, but deteriorated significantly in the finasteride group. The finasteride group experienced a greater reduction in prostate volume compared to Permixon (16% vs. 7%). Notably, mean PSA values fell significantly in the finasteride group but did not change in the Permixon group. These results suggest equivalence of the two therapies in several parameters, although the lack of a placebo arm leaves open the question of magnitude of effect.

SUMMARY

Numerous controlled trials have reported saw palmetto to be superior to placebo and possibly equivalent to the anti-androgenic agent finasteride (with fewer adverse effects) in the alleviation of nocturia, improvement of urinary flow, reduction of post-void residual bladder volume, and improvement in quality of life. However, the majority of studies have been brief (1-6 months), included small sample size, and have not employed standardized outcomes measurements. Nonetheless, the weight of available evidence favors the efficacy of saw palmetto for BPH.

References

Wilt T, Ishani A, Stark G, et al. Serenoa repens for benign prostatic hyperplasia. *Cochrane Database Syst Rev 2000;(2):CD001423.*

Wilt T, Ishani A, Mac DR. Serenoa repens for benign prostatic hyperplasia. *Cochrane Database Syst Rev 2002;(3):CD001423.*

Boyle P, Robertson C, Lowe F, et al. Meta-analysis of clinical trials of Permixon in the treatment of symptomatic benign prostatic hyperplasia. *Urology 2000;55(4):533-539.*

Mohanty NK, Jha RJ, Dutt C. Randomized double-blind controlled clinical trial of Serenoa repens versus placebo in the management of patients with symptomatic grade I to grade II benign prostatic hyperplasia (BPH). *Indian J Urol 1999;16(1):26-31.*

Gerber GS, Kuznetsov D, Johnson BC, et al. Randomized, double-blind, placebo-controlled trial of saw palmetto in men with lower urinary tract symptoms. *Urology 2001;58(6):960-964.*

Bauer HW, Casarosa C, Cosco M, et al. Saw palmetto fruit extract for treatment of benign prostatic hyperplasia. Results of a placebo-controlled double-blind study. *MMW Fortschr Med 1999;141(25):62.*

Carraro JC, Raynaud JP, Koch G, et al. Comparison of phytotherapy (Permixon) with finasteride in the treatment of benign prostate hyperplasia: a randomized international study of 1,098 patients. *Prostate 1996:29(4):231-240.*

This Herbal Medicine Fact Sheet is one of a series of fact sheets that have been produced by the Australian Centre for Complementary Medicine Education and Research (ACCMER) and the School of Population Health of the University of Queensland for GPs to use in their clinical practice. These fact sheets are currently being piloted and evaluated and if successful will be widely distributed to GPs in the future.
